# Effects of High-Quality Carbon Nanowalls Ionization-Assisting Substrates on Surface-Assisted Laser Desorption/Ionization Mass Spectrometry Performance

**DOI:** 10.3390/nano13010063

**Published:** 2022-12-23

**Authors:** Ryusei Sakai, Hiroki Kondo, Kenji Ishikawa, Takayuki Ohta, Mineo Hiramatsu, Hiromasa Tanaka, Masaru Hori

**Affiliations:** 1 Department of Electronics, Graduate School of Engineering, Nagoya University, Furo, Chikusa, Nagoya 464-8603, Japan; 2 Center for Low-Temperature Plasma Sciences, Nagoya University, Furo, Chikusa, Nagoya 464-8603, Japan; 3 Department of Electrical and Electronic Engineering, Meijo University, 1-501 Shiogamaguchi, Tempaku, Nagoya 468-8502, Japan

**Keywords:** carbon nanowalls, surface-assisted laser desorption/ionization mass spectrometry, plasma-enhanced chemical vapor deposition

## Abstract

Surface-assisted laser desorption/ionization mass spectrometry (SALDI-MS) is performed using carbon nanowalls (CNWs) for ionization-assisting substrates. The CNWs (referred to as high-quality CNWs) in the present study were grown using a radical-injection plasma-enhanced chemical vapor deposition (RI-PECVD) system with the addition of oxygen in a mixture of CH_4_ and H_2_ gases. High-quality CNWs were different with respect to crystallinity and C–OH groups, while showing similar wall-to-wall distances and a wettability comparable to CNWs (referred to as normal CNWs) grown without O_2_. The efficiency of SALDI was tested with both parameters of ion intensity and fragmental efficiency (survival yield (SY)) using N-benzylpyridinuim chloride (N-BP-CI). At a laser fluence of 4 mJ/cm^2^, normal CNWs had an SY of 0.97 and an ion intensity of 0.13, while 5-sccm-O_2_– high-quality CNWs had an SY of 0.89 and an ion intensity of 2.55. As a result, the sensitivity for the detection of low-molecular-weight analytes was improved with the high-quality CNWs compared to the normal CNWs, while an SY of 0.89 was maintained at a low laser fluence of 4 mJ/cm^2^. SALDI-MS measurements available with the high-quality CNWs ionization-assisting substrate provided high ionization and SY values.

## 1. Introduction

The method of surface-assisted laser desorption/ionization mass spectrometry (SALDI-MS) was introduced by Sunner et al. in 1995 [1]. SALDI-MS was applied for the rapid, invasive, and sensitive diagnoses of biological samples. SALDI-MS provides the advantage of a matrix-free detection method, particularly for low-molecular-weight molecules. A variety of materials have been applied as SALDI ionization-assisting substrates. For example, Si-based materials (e.g., porous Si [2] and Si nanowires (SiNWs) [3,4]), carbon-based materials (e.g., graphite [1], carbon nanotubes (CNTs) [5,6,7], graphene [7,8], carbon nanowalls (CNWs) [9,10]), and metal-based materials (e.g., Ge [11], Ag [12,13], Pt [13,14], Au [13,15,16], and TiO_2_ [17]) have been reported. The achievement of highly sensitive and nonfragmented SALDI-MS has been an issue for high-efficiency desorption/ionization without fragmentation of the analytes [18]. N-benzylpyridinium chloride (N-BP-Cl) has been used as a chemical thermometer to indicate the degree of ionization fragmentation [19]. The survival yield (SY) of N-BP-Cl is defined using the intensities of benzylpyridinium ions ((BP)^+^) and benzylium ions ((C_7_H_7_)^+^). The previously reported SY values were 0.97 for CNWs grown without O_2_ (referred to as normal CNWs) at a laser fluence of 4 mJ/cm^2^ [10], 0.8 for SiNWs at as laser fluence of 4 mJ/cm^2^ [4], 0.97 for perfluorodecyltrichlorosilane-modified platinum nanoflowers (FDTS-PtNFs) with a citrate buffer at approximately 10 mJ/cm^2^ [14], and 0.964 ± 0.023 for CNTs at 25 mJ/cm^2^ [7]. SALDI ionization-assisting substrates with unique morphologies, such as SiNWs, PtNFs, and CNWs, enable desorption/ionization at low laser fluences [7,10,14]. Carbon-based materials, such as CNTs, also provide excellent SALDI properties due to their optical properties, chemical termination, and phase transition/destruction [7]. It has been reported that improved crystallinity and the introduction of carboxyl groups for oxidized CNTs [6] and oxidized graphitized carbon black nanoparticles (GCB) [20] enhanced the signal intensities in SALDI measurements. Compared to other ionization-assisting substrates, CNW ionization-assisting substrates, which are comprised of carbon and have unique morphologies, offer two advantages. One is that CNWs achieved desorption/ionization at the lowest laser fluence of 4 mJ/cm^2^ [10], and the other is that at the lowest laser fluence of 4 mJ/cm^2^, CNWs grown without oxygen achieved a higher SY of 0.97 [10] than an SY of 0.8 for SiNWs [4].

Hori et al. invented SALDI-MS with CNWs as SALDI ionization-assisting substrates [21,22]. CNWs are vertically oriented wall-like structures that are comprised of multiple stacks of graphene sheets [23,24]. CNWs are grown on various substrates, such as Si, without a catalyst [23]. CNWs have a high density of graphene edges formed by graphene with excellent optical properties at the top edge [25]. CNWs retain their structure in bio-liquids and can be applied to cell culture plates [26]. The morphology of CNWs, such as the wall density, can be changed by altering the plasma during growth [27,28]. The oxygen-added growth of CNWs alters their physical properties, such as their crystallinity and electrical conductivity [29,30]. O radicals enhance the nucleation of CNWs due to a decrease in amorphous carbon during the initial growth of the CNWs. O radicals also suppress branching of the sides of CNWs during growth. The hydrophilic and hydrophobic properties of CNWs can easily be changed using atmospheric pressure plasma [31]. These characteristics make CNWs a promising SALDI ionization-assisting substrate. First, when irradiated with a high-intensity laser, high optical absorption and tip-edge structures of CNWs possibly form an electric field that assists with the ionization of molecules located on the surface [9,10]. Second, rapid SALDI-MS can be performed by simply dropping a bio-liquid onto the CNW ionization-assisting substrates. For example, dispersion and spots need to be considered before dropping a bio-liquid due to its aggregation in other carbon nanomaterials ionization-assisting substrates, such as CNTs [7]. Third, the preparation of CNW ionization-assisting substrates is more practical with respect to growth conditions. For example, CNT growth requires a catalyst, while no catalyst is needed for CNW growth [23]. By controlling the wall density over a wide range from 142 to 467 nm, CNWs with a wall-to-wall distance of 142 nm were determined as effective for SALDI, and resulted in low fragmentation and high ion mass signal intensities for the analyte molecules [10]. Hydrophilic CNWs are more effective for the detection of small biomolecules [9]. Whereas optimizations of CNWs for SALDI-MS have been reported, the elucidation of desorption/ionization mechanisms in CNWs has been limited. It is yet to be clarified whether specific properties of CNWs (e.g., crystallinity, electrical conductivity, etc.) can affect SALDI properties such as the ionization efficiency and SY. 

In this study, the effect of CNWs grown with the introduction of oxygen (referred to as high-quality CNWs) on the properties of SALDI was investigated in detail. High-quality CNWs were grown in a radical-injection plasma-enhanced chemical vapor deposition (RI-PECVD) system by the addition of oxygen during CNW growth [24,29,30,32]; the morphology and physical properties of CNWs change when the growth conditions include the addition of oxygen. Considering a previous report that the morphology of CNWs affected SALDI properties [10], high-quality CNWs were grown with a small amount of oxygen addition within the range that did not affect the morphology as much as possible. The performance of high-quality CNWs as ionization-assisting substrates was tested with respect to the ionization intensity and fragmental efficiency in SALDI measurements of N-benzylpyridinuim chloride (N-BP-CI).

## 2. Materials and Methods

### 2.1. Preparation of CNWs as Ionization-Assisting Substrates

**Figure 1:**
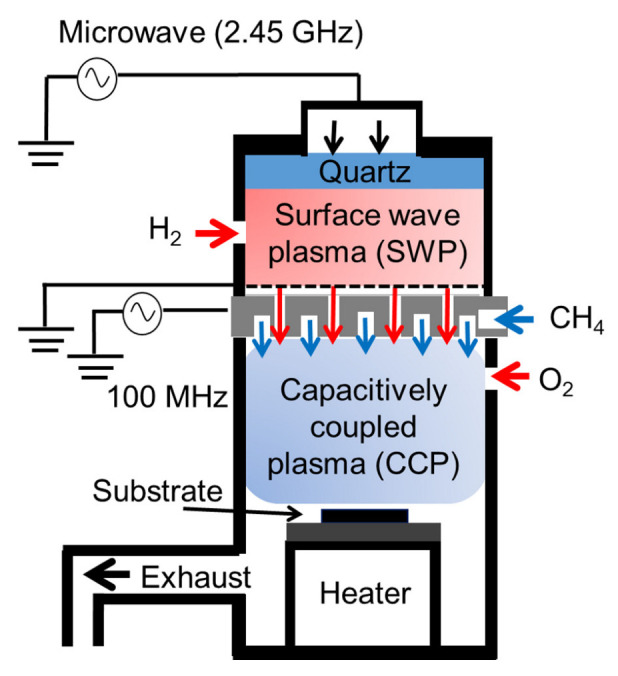
Radical-injection plasma-enhanced chemical vapor deposition (RI-PECVD) system with CH_4_, H_2_, and O_2_ gases.

CNW ionization-assisting substrates were grown on Si substrates with an RI-PECVD system (Katagiri Engineering Co., Ltd., Kanagawa, Japan) [24,29,30,32]. Two types of plasma source were used in this system to precisely and independently generate and control radical species, as shown in Figure 1. One was a surface-wave plasma (SWP) source located at an upper region, and the other was a capacitively coupled plasma (CCP) source. The two plasma sources were connected through a mesh electrode near an upper electrode for the CCP. A microwave (2.45 GHz) power of 400 W was used for the SWP. A radio frequency (RF) power of 100 MHz and 400 W was applied for the CCP, and the distance between the CCP upper electrode and the lower stage was 30 mm. The total pressure in the chamber was kept at 1 Pa. The temperature of the substrates was kept at approximately 650 °C during the entire deposition process.

Normal CNWs were grown under the following conditions. During plasma generation, 100 sccm of CH_4_ and 50 sccm of H_2_ gases were introduced into the CCP and SWP chambers, respectively. The deposition time for CNW growth was 290 s.

High-quality CNWs were grown through the addition of O_2_ gas and with the same growth conditions as those for the normal CNWs. A small amount of oxygen was added, aiming for no change in wall density, because the wall density of CNWs affects SALDI performance [10]. High-quality CNWs were grown with a deposition time of 320 s for an oxygen flow rate of 5 sccm and 380 s for an oxygen flow rate of 10 sccm to achieve a CNW height of approximately 500 nm. The crystallinity of the CNWs was previously reported to be improved when O_2_ gas with a flow rate of 5 or 10 sccm was introduced into the CCP source [29,30].

In the present experiment, CNWs that were at least 14 days old after growth were used because it has been reported that the CNW edges initially contain many defects and the surfaces become highly hydrophobic over time with inert and stable terminations [33].

The morphology of the CNWs was observed using scanning electron microscopy (SEM; Hitachi High Technologies Corporation, Tokyo, Japan, SU8230). The average wall-to-wall distance was defined as the length of a straight line divided by the number of walls crossing the line in the top view images. Raman spectra of the CNWs were acquired using a laser with a wavelength of 532 nm (Renishaw plc, Wotton-under-Edge, UK, inVia Raman). The chemical bonding states of the CNW surfaces in the C1s and O1s regions were analyzed using X-ray photoelectron spectroscopy (XPS; Vacuum Generator Scientific, Waltham, MA, USA, ESCAlab 250i). The wettability of the CNWs was investigated with contact angle measurements using three different solutions of deionized water (Milli-Q), methylene iodide (Kishida Chemical Co. Ltd., Osaka, Japan, 000-24382), and ethylene glycol (Kishida Chemical Co. Ltd., Osaka, Japan, 000-29332). The volume of the solution droplets was set to 1 mL.

### 2.2. CNWs-SALDI-TOF-MS

Laser light was provided by the fourth harmonic wave with a wavelength of 266 nm from a Nd:YAG laser (repetition rate: 30 Hz, pulse width: 2 ns; Spectra-Physics, Quanta-Ray Pro 250). The laser light was focused on the CNWs/Si sample surface located at the ion collection point of a time-of-flight mass spectrometer system (TOF-MS; Toyama Co., Ltd., Kanagawa, Japan) [9,10]. The background pressure of the ionization chamber was kept below 10 ^− 7^ Pa. The ion signals were detected in positive-ion mode using a microchannel plate (MCP) with a high voltage of 2 kV applied. The incident laser fluences were set to 4, 8, and 12 mJ/cm^2^ for the detection of N-BP-Cl. Details of CNWs-SALDI-TOF-MS have been previously published [9,10].

N-BP-Cl (C_12_H_12_ClN; Alfa Chemistry, Stony Brook, NY, USA, ACM2876133, 204 Da) was used to evaluate the degree of fragmentation in the desorption/ionization process [7,10,13]. N-BP-Cl was dissolved in methanol (Fuji Film Wako Pure Chemical Corporation, Osaka, Japan, 138-06473). The concentration was adjusted to 0.1 mM with reference to previous literature [7,10]. A total of 5 µL of the N-BP-Cl solution was then dropped onto the CNW ionization-assisting substrate and left to dry in the ambient air. The SY of N-BP-Cl is defined as:

(1) $$\mathrm{SY}=\left(\frac{I_{M}}{I_{M}+I_{F}}\right)$$

where I_M_ and I_F_ are the signal intensities for benzylpyridinium ions ((BP)^+^) with *m/z* of 170, and benzylium ions ((C_7_H_7_)^+^) with *m/z* of 91, respectively [7,10,13]. When (BP)^+^ is desorbed/ionized, the C–N bond between the benzyl and pyridine is simply cleaved, producing fragment ions. For more details, please refer to the publication on the SY method [19].

## 3. Results and Discussion

### 3.1. Physicochemical Observation of the Normal and High-Quality CNWs

**Figure 2:**
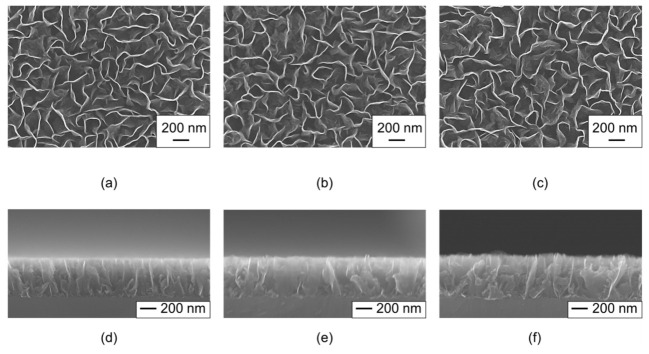
Scanning electron microscopy (SEM) images for (**a**–**c**) top and (**d**–**f**) cross sections of the (**a**,**d**) normal CNWs, (**b**,**e**) 5-sccm-O_2_– and (**c**,**f**) 10-sccm-O_2_– high-quality CNWs.

The morphology of the CNWs was observed by acquiring top and cross-sectional SEM images, as shown in Figure 2. Figure 2a shows the morphology of the normal CNWs. The wall-to-wall distance for normal CNWs was 142 ± 3.7 nm. Figure 2b,c show the morphology of the high-quality CNWs, where the wall-to-wall distances for the CNWs were 152 ± 6.2 nm for an oxygen flow rate of 5 sccm and 157 ± 6.6 nm for an oxygen flow rate of 10 sccm. The height of all CNWs was approximately 500 nm, as shown in Figure 2d–f.

**Figure 3:**
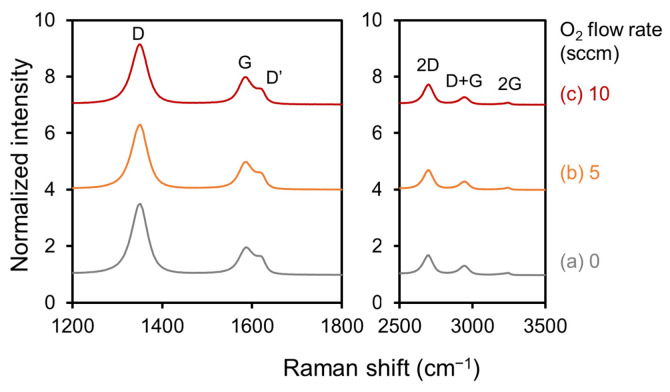
Raman spectra of (**a**) normal CNWs, (**b**) 5-sccm-O_2_–, and (**c**) 10-sccm-O_2_– high-quality CNWs.

Figure 3 shows a series of Raman spectra that were normalized with respect to the peak intensity of the 1350 cm^− 1^ D-band. The observed Raman peaks were identified as being due to graphene materials. The peaks at 1586 cm^− 1^ were attributed to the six-membered ring structure of graphene, i.e., the G-band. The D-band was due to disorders and defects of the six-membered ring structures. The peak at 1620 cm^− 1^ was assigned as the D’-band, which is related to the finite size of the graphite crystallites and their edges. If the peak intensities for the D- and D’-bands are large with respect to the G band, then the CNWs are assumed to have defects in the six-membered ring structures and graphene edges. In addition, it has been reported that graphene-based materials with a large amount of graphene edges have a 2D-band at approximately 2690 cm^− 1^, a D+G-band at approximately 2940 cm^− 1^, and a 2G-band at approximately 3200 cm^− 1^. The results indicated that all the CNWs had almost the same characteristics, regardless of the introduction of oxygen [27].

**Figure 4:**
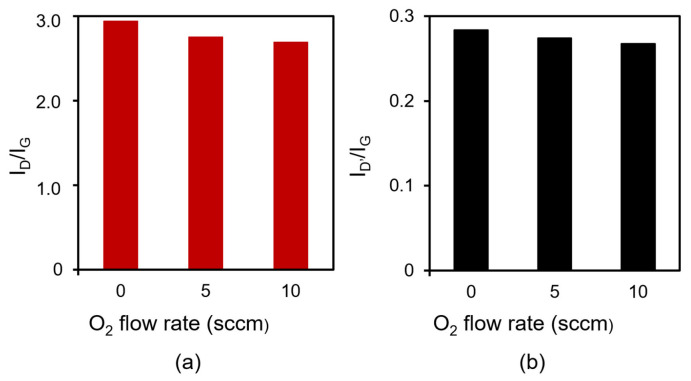
Peak area intensity ratios for (**a**) the D-band to G-band peaks (I_D_/I_G_) and (**b**) those for the D’-band to G-band peaks (I_D’_/I_G_) in Raman spectra for normal CNWs, 5-sccm-O_2_–, and 10-sccm-O_2_– high-quality CNWs.

Figure 4 shows peak area intensity ratios for the D-band to G-band peaks (I_D_/I_G_) and those for the D’-band to G-band peaks (I_D’_/I_G_). For example, I_D_/I_G_ were 2.94, 2.75, and 2.69 with O_2_ flow rates of 0, 5, and 10 sccm, respectively. I_D_/I_G_ and I_D’_/I_G_ decreased when the O_2_ flow rate was increased, which indicates that the addition of oxygen allowed the growth of more crystalline CNWs, as previously reported [29,30]. The addition of O_2_ gas was assumed to decrease amorphous carbon and nucleation during the initial growth of the CNWs. In addition, CNWs grown with oxygen in this study (high-quality CNWs) may have improved the electrical conductivity and formed monolithic graphene sheets, similar to CNWs grown with oxygen, as previously reported [30].

**Figure 5:**
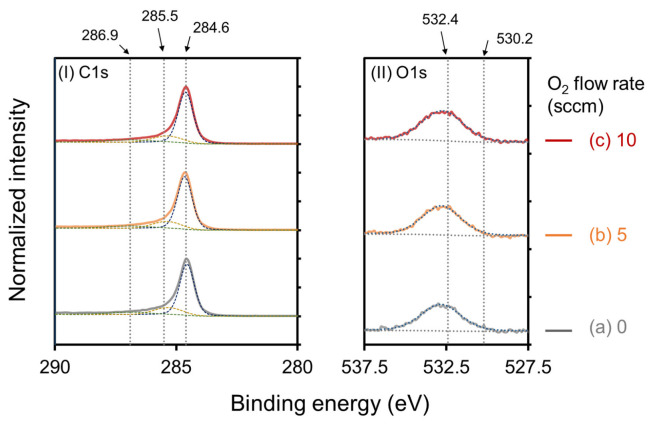
(**I**) C1s and (**II**) O1s XPS spectra of (**a**) normal CNWs, (**b**) 5-sccm-O_2_–, and (**c**) 10-sccm-O_2_– high-quality CNWs. The C1s spectra and O1s spectra were normalized with respect to the peak intensities for sp^2^ C–C bonds at 284.6 eV.

Figure 5 shows (I) C1s and (II) O1s XPS spectra of the CNWs. The C1s and O1s spectra were normalized with respect to the peak intensities for sp^2^ C–C bonds at 284.6 eV. CNWs grown at O_2_ flow rates of (a) 0, (b) 5, and (c) 10 sccm are shown in Figure 5. The C1s peak was decomposed into three components centered at 284.6, 285.5, and 286.9 eV (Figure 5I). The main C1s peak at 284.6 eV corresponded to sp^2^ C-C bonds of graphite [27,34]. The peak centered at 285.5 eV originated from sp^3^-bonded carbon atoms (sp^3^ C-C), and the peak at 286.9 eV was due to C-O-C bonds [34]. The O1s peak was also observed regardless of the oxygen flow rate (Figure 5II). It has been reported that O1s is formed by the C-OH peak at 532.4 eV and the C=O peak at 530.2 eV [27]. All CNWs exhibited surface-bonding states of typical CNWs [27,34]. The XPS survey spectra was shown in Figure S1 in the Supplementary Materials.

**Figure 6:**
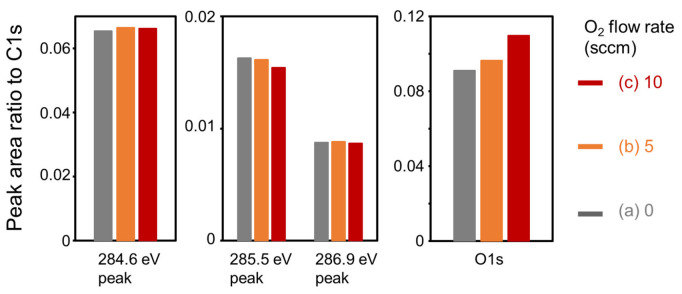
Ratios of each peak area to the total area of C1s for the (**a**) normal CNWs, and the high-quality CNWs with oxygen flow rates of (**b**) 5 sccm and (**c**) 10 sccm.

Figure 6 shows the peak area ratios of the 284.6 eV peak to C1s, 285.5 eV peak to C1s, 286.9 eV peak to C1s, and O1s to C1s for the CNWs. The ratios of the 284.6 eV XPS peak area to the total peak area of C1s and the total O1s area to the total peak area of C1s increased with the oxygen flow rate. The ratio of the 285.5 eV peak area to the total peak area of C1s decreased with an increase in the oxygen flow rate. The ratio of the 286.9 eV peak area to the total peak area of C1s showed no dependence on the oxygen flow rate. These results indicate that sp^2^ C-C bonds at 284.6 eV and C-OH at 532.4 eV increased instead of decreasing the sp^3^ C-C bond. Thus, graphene sheets forming the high-quality CNWs were more graphene-like, i.e., the high-quality CNWs had high crystallinity.

**Figure 7:**
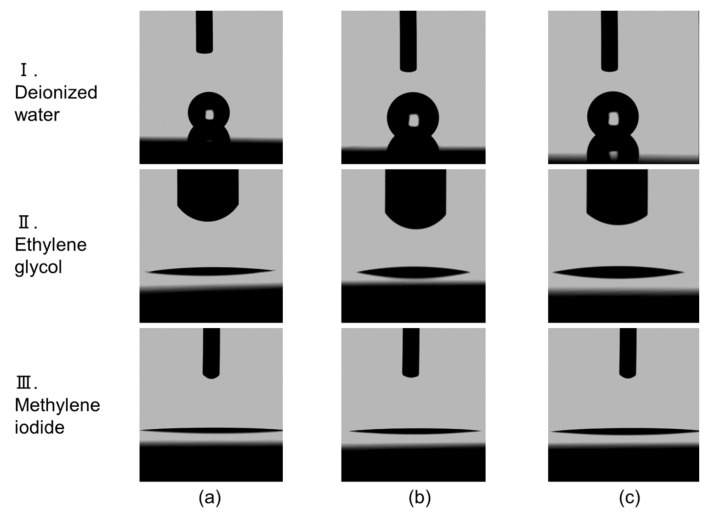
Optical photographs of droplet impressions taken during contact angle measurements on CNWs surfaces grown with various oxygen flow rates**.** (**a**) Normal CNWs, (**b**) 5-sccm-O_2_–, and (**c**) 10-sccm-O_2_– high-quality CNWs. Row (I) deionized water, (II) ethylene glycol, and (III) methylene iodide.

Figure 7 shows optical photographs of droplet impressions for (I) deionized water, (II) ethylene glycol, and (III) methylene iodide, taken during contact angle measurements on CNW surfaces grown with various oxygen flow rates of (a) normal CNWs, (b) 5-sccm-O_2_–, and (c) 10-sccm-O_2_– high-quality CNWs.

**Figure 8:**
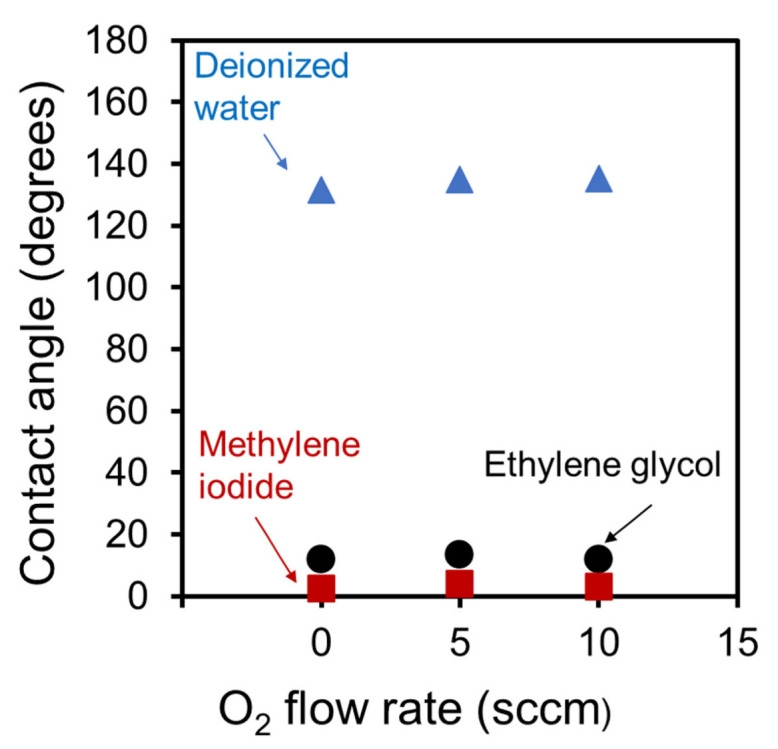
Contact angles for each droplet on the surfaces of CNWs grown at various oxygen flow rates.

Figure 8 shows the contact angles for each droplet on CNW surfaces grown at different oxygen flow rates. Contact angles were similarly obtained for deionized water, ethylene glycol, and methylene iodide on CNW surfaces grown at various oxygen flow rates. The results indicate that the wettability of the CNW surfaces grown with all oxygen flow rates was not significantly affected by the crystallinity. As described in Section 2.1, the surface condition of the CNWs was stable and hydrophobic because the CNWs were more than 14 days old, which was consistent with previous research [33]. It has been reported that differences in wettability affect the sensitivity for detection [9]. This suggests that the addition of oxygen does not affect wettability, i.e., it does not affect the SALDI sensitivity.

### 3.2. SALDI Performance of the High-Quality CNWs

**Figure 9:**
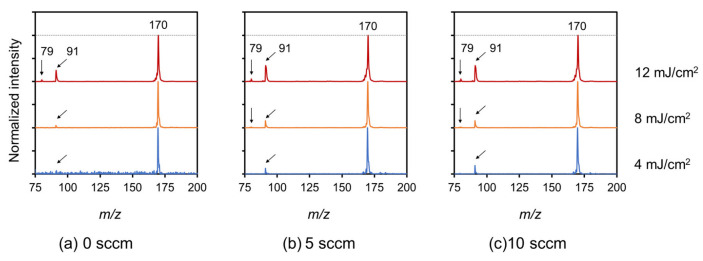
SALDI mass spectra of N-BP-Cl on high-quality CNWs ionization-assisting substrates measured at various laser fluences of 4, 8, and 12 mJ/cm^2^. (**a**) Normal CNWs, (**b**) 5-sccm-O_2_–, and (**c**)10-sccm-O_2_– high-quality CNWs. **(a)** Normal CNWs are cited from the publication for comparison [10].

Figure 9 shows the SALDI mass spectra of N-BP-Cl on high-quality CNWs ionization-assisting substrates. The laser fluences were set at 4, 8, and 12 mJ/cm^2^. All spectra were normalized and shown with respect to the intensity at *m*/*z* = 170. Two characteristic signals related to N-BP-Cl were observed. One indicated (BP)^+^ ions at *m/z* = 170 and the other (C_7_H_7_)^+^ ions at *m*/*z* = 91 [7,10,13]. In addition, relatively small peaks due to pyridine ions ((C_5_H_5_N)^+^) also appeared at *m/z* = 79 in some spectra [10]. For the high-quality CNWs, the (C_7_H_7_)^+^ ions peak at *m/z* = 91, which corresponded to fragmentation, became larger. On the other hand, the spectra of high-quality CNWs (Figure 9b,c) at 4 mJ/cm^2^ were more clearly observed than in the case of normal CNWs (Figure 9a).

**Figure 10:**
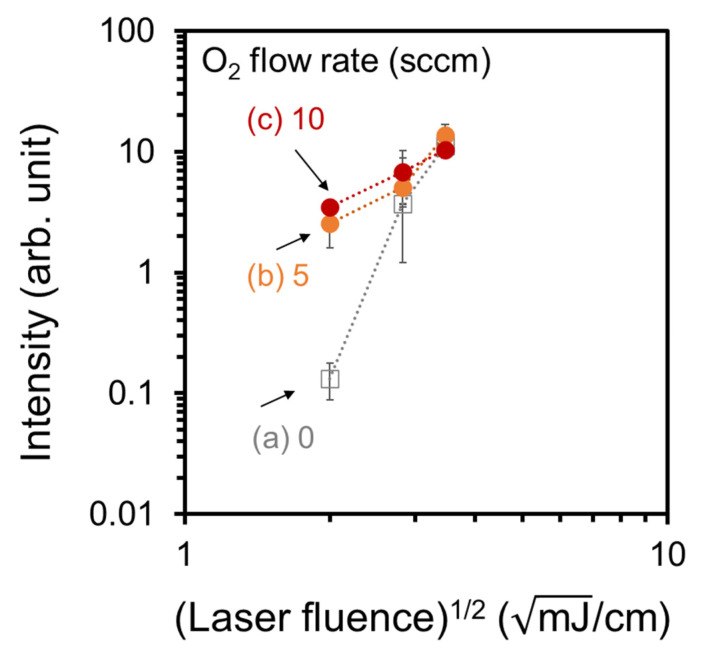
Ion intensity for N-BP-Cl on the high-quality CNWs ionization-assisting substrates as a function of the laser fluence: (**a**) normal CNWs, (**b**) 5-sccm-O_2_–, and (**c**) 10-sccm-O_2_– high-quality CNWs.

Figure 10 shows the ion intensities for N-BP-Cl on the normal and high-quality CNWs ionization-assisting substrates as a function of the laser fluence. The ion intensity was defined as the sum of the peak intensities at *m/z* = 170 and 91. The ion intensities measured using the CNW ionization-assisting substrates increased with the laser fluence. At a low laser fluence of 4 mJ/cm^2^, the ion intensity for the high-quality CNWs was clearly higher than that for the normal CNWs.

The rate of increase in ion intensity as a function of the laser fluence was different for normal CNWs grown without O_2_ and high-quality CNWs grown with O_2_. As explained in Section 3.1, physicochemical observations of high-quality and normal CNWs showed that the main difference was the crystallinity and the C-OH groups. Therefore, from these results, it can be assumed that the crystallinity and the C-OH bond of the CNWs had an influence.

Why did high-quality CNWs with high crystallinity and C–OH groups enhance the ion intensity? One mechanism could be that the monolithic graphene sheets that form the high-quality CNWs contributed to a further enhancement of the electric field on the CNWs’ edge. We have previously indicated that the factor influencing ionization of the measured molecules on CNWs is the electric field concentration at the graphene edges of the CNWs [10]. The O radicals produced during the growth of high-quality CNWs etch the amorphous carbon and suppress branching of the CNWs, which allows for higher crystallinity and electrical conduction [29,30]. The more graphene-like, less branched CNWs, i.e., high-quality CNWs, can enhance the electric field on the edges compared to the normal CNWs, which contributes to the improved ion intensity.

Another mechanism could be the C–OH groups on the surface of high-quality CNWs. It has been reported that the signal was enhanced in oxidized CNTs [6] and GCB [20], mainly due to the increase in C–OH groups. However, it is not sufficiently clear how the structure and electrical properties of the individual graphene sheets of CNWs differ between the normal and high-quality CNWs. More detailed studies, such as using X-ray diffraction, are, thus, required to clarify how these features affect the ionization properties.

**Figure 11:**
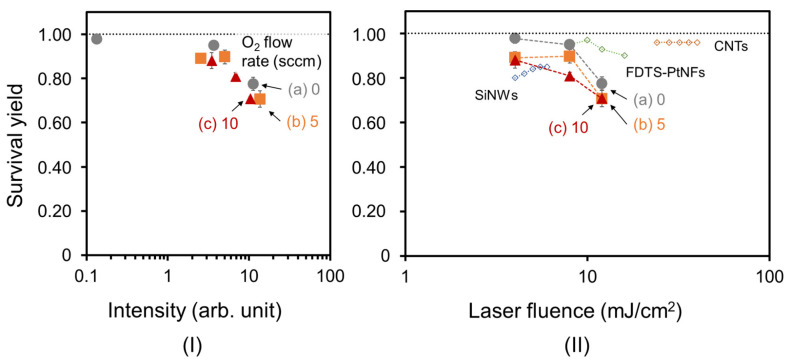
SY values for N-BP-Cl on high-quality CNWs ionization-assisting substrates as a function of (**I**) the ion intensity and (**II**) laser fluence**:** (**a**) normal CNWs, (**b**) 5-sccm-O_2_–, and (**c**) 10-sccm-O_2_– high-quality CNWs. For each CNW sample, the increase in the ion intensity corresponds to laser fluences of 4, 8, and 12 mJ/cm^2^, in that order. SY values of (**a**) normal CNWs [10], SiNWs [4], FDTS-PtNFs with a citrate buffer [14], and CNTs [13] are cited for comparison, respectively.

Figure 11(I) shows SY values for N-BP-Cl on normal and high-quality CNW ionization-assisting substrates as a function of the ion intensity. For each CNW substrate, the increase in the ion intensity corresponded to the laser fluences of 4, 8, and 12 mJ/cm^2^, in that order. Here, the SY values for the normal CNWs were retrieved from the publication [10]. For example, in the case of a laser fluence of 4 mJ/cm^2^ (the lowest ion intensity for each CNW substrate), the average SY values were 0.97, 0.89, and 0.88 for the CNW ionization-assisting substrates with normal CNWs, 5-sccm-O_2_–, and 10-sccm-O_2_– high-quality CNWs, respectively. The SY values for the high-quality CNWs decreased as the oxygen flow increased.

Figure 11(II) shows the SY values for N-BP-Cl on high-quality CNW ionization-assisting substrates as a function of laser fluence. Here, the results of the previous study and the SY of the present study are compared. The SY value for SiNWs was reported to be approximately 0.8 under a laser irradiation of 4 mJ/cm^2^ [4]. An SY value of 0.97 for FDTS-PtNFs with a citrate buffer was also reported at a laser fluence of approximately 10 mJ/cm^2^ [14]. We also reported a high SY value of 0.97 for normal CNWs, which is close to 1, at the lowest laser fluence of 4 mJ/cm^2^ [10]. Compared to these reported high SY values, the SY values of 0.89 and 0.88 for 5-sccm-O_2_- and 10-sccm-O_2_– high-quality CNWs at a laser fluence of 4 mJ/cm^2^ were comparable. High-quality CNW ionization-assisting substrates grown with oxygen also offered the same two advantages as normal CNW ionization-assisting substrates grown without oxygen, as described in Chapter 1. This means that the electric field at the graphene edge of CNWs with optimized wall-to-wall distances may cause more efficient desorption/ionization, as discussed in the publication [10].

As a result, at a laser fluence of 4 mJ/cm^2^ (the lowest value of ion intensity shown in Figure 11(I)), the SY value for the high-quality CNWs decreased compared to that for the normal CNWs, but the ion intensity increased. For example, at a laser fluence of 4 mJ/cm^2^, the normal CNWs had an SY value of 0.97 and an ion intensity of 0.13, while the 5-sccm-O_2_– high-quality CNWs had an SY value of 0.89 and an ion intensity of 2.55. This indicates that the higher crystallinity of CNWs with the same surface morphology can increase the ionization intensity, while having a slightly negative effect on the soft ionization properties, such as the SY. It was assumed that excessive crystallinity results in the formation of a strong electric field that assists ionization. This means that the high-quality CNWs with moderate crystallinity clearly increased the ion intensity while softly ionizing the N-BP-Cl. High-quality CNW ionization-assisting substrates could, thus, be available for mass-spectrometry analysis of samples, such as biomolecules, while maintaining high soft ionization ability.

## 4. Conclusions

The laser desorption/ionization properties of high-quality CNWs grown by the introduction of oxygen were investigated in detail. The high-quality CNWs showed high crystallinity and an increase in C–OH groups, while showing wall-to-wall distances and wettability comparable to normal CNWs grown without oxygen. At a laser fluence of 4 mJ/cm^2^, the normal CNWs had an SY value of 0.97 and ion intensity of 0.13, while 5-sccm-O_2_– high-quality CNWs had an SY value of 0.89 and ion intensity of 2.55. The SY values were decreased for the high-quality CNWs compared to normal CNWs, but the ion intensity was increased. The results presented here indicate that high-quality CNWs had higher ionization efficiency while suppressing the fragmentation in desorption/ionization, i.e., they exhibited high SY values.

## Data Availability

The data presented in this study are available on request from the corresponding author.

## Supplementary Materials

The following supporting information can be downloaded at: https://www.mdpi.com/article/10.3390/nano13010063/s1, Figure S1: (I) the entire and (II) enlarged XPS spectra of (a) normal CNWs, (b) 5-sccm-O_2_–, and (c) 10-sccm-O_2_– high-quality CNWs. XPS spectra were normalized with respect to the C1s peak intensities;

## References

[1] Sunner J., Dratz E., Chen Y.-C. (1995). Graphite surface-assisted laser desorption/ionization time-of-flight mass spectrometry of peptides and proteins from liquid solutions. Anal. Chem..

[2] Wei J., Buriak J.M., Siuzdak G. (1999). Desorption–ionization mass spectrometry on porous silicon. Nature.

[3] Go E.P., Apon J.V., Luo G., Saghatelian A., Daniels R.H., Sahi V., Dubrow R., Cravatt B.F., Vertes A., Siuzdak G. (2005). Desorption/Ionization on Silicon Nanowires. Anal. Chem..

[4] Luo G., Chen Y., Daniels H., Dubrow R., Vertes A. (2006). Internal Energy Transfer in Laser Desorption/Ionization from Silicon Nanowires. J. Phys. Chem. B.

[5] Xu S., Li Y., Zou H., Qiu J., Guo A.Z., Guo B. (2003). Carbon Nanotubes as Assisted Matrix for Laser Desorption/Ionization Time-of-Flight Mass Spectrometry. Anal. Chem..

[6] Pan C., Xu S., Hu L., Su X., Ou J., Zou H., Guo Z., Zhang Y., Guo B. (2005). Using oxidized carbon nanotubes as matrix for analysis of small molecules by MALDI-TOF MS. J. Am. Soc. Mass Spectrom..

[7] Tang H.-W., Ng K.-M., Lu W., Che C.-M. (2009). Ion Desorption Efficiency and Internal Energy Transfer in Carbon-Based Surface-Assisted Laser Desorption/Ionization Mass Spectrometry: Desorption Mechanism(s) and the Design of SALDI Substrates. Anal. Chem..

[8] Dong X., Cheng J., Li J., Wang Y. (2010). Graphene as a Novel Matrix for the Analysis of Small Molecules by MALDI-TOF MS. Anal. Chem..

[9] Ohta T., Ito H., Ishikawa K., Kondo H., Hiramatsu M., Hori M. (2019). Atmospheric Pressure Plasma-Treated Carbon Nanowalls’ Surface-Assisted Laser Desorption/Ionization Time-of-Flight Mass Spectrometry (CNW-SALDI-MS). J. Carbon Res..

[10] Sakai R., Ichikawa T., Kondo H., Ishikawa K., Shimizu N., Ohta T., Hiramatsu M., Hori M. (2021). Effects of Carbon Nanowalls (CNWs) Substrates on Soft Ionization of Low-Molecular-Weight Organic Compoundsin Surface-Assisted Laser Desorption/Ionization Mass Spectrometry (SALDI-MS). Nanomaterials.

[11] Seino T., Sato H., Yamamoto A., Nemoto A., Torimura M., Tao H. (2007). Matrix-Free Laser Desorption/Ionization-Mass Spectrometry Using Self-Assembled Germanium Nanodots. Anal. Chem..

[12] Cioffi N., Colaianni L., Pilolli R., Calvano C.D., Palmisano F., Zambonin P.G. (2009). Silver nanofractals: Electrochemical synthesis, XPS characterization and application in LDI-MS. Anal. Bioanal. Chem..

[13] Ng K.-M., Chau S.-L., Tang H.-W., Wei X.-G., Lau K.-C., Ye F., Ng A.M.-C. (2015). Ion-Desorption Efficiency and Internal-Energy Transfer in Surface-Assisted Laser Desorption/Ionization: More Implication(s) for the Thermal-Driven and Phase-Transition-Driven Desorption Process. J. Phys. Chem. C.

[14] Kawasaki H., Yonezawa T., Watanabe A.T., Arakawa R. (2007). Platinum Nanoflowers for Surface-Assisted Laser Desorption/Ionization Mass Spectrometry of Biomolecules. J. Phys. Chem. C.

[15] McLean J.A., Stumpo K.A., Russell D.H. (2005). Size-Selected (2−10 nm) Gold Nanoparticles for Matrix Assisted Laser Desorption Ionization of Peptides. J. Am. Chem. Soc..

[16] Pilolli R., Palmisano F., Cioffi N. (2012). Gold nanomaterials as a new tool for bioanalytical applications of laser desorption ionization mass spectrometry. Anal. Bioanal. Chem..

[17] Lee K.-H., Chiang C.-K., Lin Z.-H., Chang H.-T. (2007). Determining enediol compounds in tea using surface-assisted laser desorption/ionization mass spectrometry with titanium dioxide nanoparticle matrices. Rapid Commun. Mass Spectrom..

[18] Picca R.A., Calvano C.D., Cioffi N., Palmisano F. (2017). Mechanisms of Nanophase-Induced Desorption in LDI-MS. A Short Review. Nanomaterials.

[19] Greisch J.-F., Gabelica V., Remacle F., De Pauw E. (2003). Thermometer ions for matrix-enhanced laser desorption/ionization internal energy calibration. Rapid Commun. Mass Spectrom..

[20] Amini N., Shariatgorji M., Thorsén G. (2009). SALDI-MS Signal enhancement using oxidized graphitized carbon black nanoparticles. J. Am. Soc. Mass Spectrom..

[21] Hori M., Sato H., Toyoshima Y., Hiramatsu M. (2009). Sample Substrate for Laser Desorption Ionization-Mass Spectrometry, and Method and Device both Using the Same for Laser Desorption Ionization-Mass Spectrometry. Japan Patent.

[22] Hori M., Sato H., Toyoshima Y., Hiramatsu M. (2013). Sample Substrate for Laser Desorption Ionization-Mass Spectrometry, and Method and Device both Using the Same for Laser Desorption Ionization-Mass Spectrometry. U.S. Patent.

[23] Hiramatsu M., Shiji K., Amano H., Hori M. (2004). Fabrication of vertically aligned carbon nanowalls using capacitively coupled plasma-enhanced chemical vapor deposition assisted by hydrogen radical injection. Appl. Phys. Lett..

[24] Hiramatsu M., Hori M. (2010). Carbon Nanowalls.

[25] Kawai S., Kondo S., Takeuchi W., Kondo H., Hiramatsu M., Hori M. (2010). Optical Properties of Evolutionary Grown Layers of Carbon Nanowalls Analyzed by Spectroscopic Ellipsometry. Jpn. J. Appl. Phys..

[26] Watanabe H., Kondo H., Okamoto Y., Hiramatsu M., Sekine M., Baba Y., Hori M. (2014). Carbon nanowall scaffold to control culturing of cervical cancer cells. Appl. Phys. Lett..

[27] Cho H.J., Kondo H., Ishikawa K., Sekine M., Hiramatsu M., Hori M. (2014). Density control of carbon nanowalls grown by CH4/H2 plasma and their electrical properties. Carbon.

[28] Ichikawa T., Shimizu N., Ishikawa K., Hiramatsu M., Hori M. (2020). Synthesis of isolated carbon nanowalls via high-voltage nanosecond pulses in conjunction with CH4/H2 plasma enhanced chemical vapor deposition. Carbon.

[29] Kondo S., Kawai S., Takeuchi W., Yamakawa K., Den S., Kano H., Hiramatsu M., Hori M. (2009). Initial growth process of carbon nanowalls synthesized by radical injection plasma-enhanced chemical vapor deposition. J. Appl. Phys..

[30] Takeuchi W., Takeda K., Hiramatsu M., Tokuda Y., Kano H., Kimura S., Sakata O., Tajiri H., Hori M. (2010). Monolithic self-sustaining nanographene sheet grown using plasma-enhanced chemical vapor deposition. Phys. Status Solidi A.

[31] Watanabe H., Kondo H., Sekine M., Hiramatsu M., Hori M. (2012). Control of Super Hydrophobic and Super Hydrophilic Surfaces of Carbon Nanowalls Using Atmospheric Pressure Plasma Treatments. Jpn. J. Appl. Phys..

[32] Kondo S., Hori M., Yamakawa K., Den S., Kano H., Hiramatsu M. (2008). Highly reliable growth process of carbon nanowalls using radical injection plasma-enhanced chemical vapor deposition. J. Vac. Sci. Technol. B Microelectron. Nanometer Struct. Process. Meas. Phenom..

[33] Vizireanu S., Ionita M.D., Ionita R.E., Stoica S.D., Teodorescu C.M., Husanu M.A., Apostol N.G., Baibarac M., Panaitescu D., Dinescu G. (2017). Aging phenomena and wettability control of plasma deposited carbon nanowall layers. Plasma Process. Polym..

[34] Santhosh N.M., Filipič G., Kovacevic E., Jagodar A., Berndt J., Strunskus T., Kondo H., Hori M., Tatarova E., Cvelbar U. (2020). N-Graphene Nanowalls via Plasma Nitrogen Incorporation and Substitution: The Experimental Evidence. Nano-Micro Lett..

