# Supplementary material for: Effects of High-Quality Carbon Nanowalls Ionization-Assisting Substrates on Surface-Assisted Laser Desorption/Ionization Mass Spectrometry Performance
Source: Nanomaterials (Basel). 2022 Dec 23;13(1):63. doi: 10.3390/nano13010063 (PMC9823508; doi:10.3390/nano13010063)
Supplement: Supplementary file 1 [file nanomaterials-13-00063-s001.zip › nanomaterials-2070441-supplementary.pdf]

nanomaterials

Supporting Information

Effects of High-Quality Carbon Nanowalls Ionization-Assisting Substrates on Surface-Assisted Laser Desorption/Ionization Mass Spectrometry Performance

Ryusei Sakai [1], Hiroki Kondo [2], Kenji Ishikawa [2,*], Takayuki Ohta [3], Mineo Hiramatsu [3], Hiromasa Tanaka [2] and Masaru Hori [2]

1 Department of Electronics, Graduate School of Engineering, Nagoya University, Furo, Chikusa, Nagoya 464-8603, Japan
2 Center for Low-temperature Plasma Sciences, Nagoya University, Furo, Chikusa, Nagoya 464-8603, Japan
3 Department of Electrical and Electronic Engineering, Meijo University, 1-501 Shiogamaguchi, Tempaku, Nagoya 468-8502, Japan
* Correspondence: ishikawa@plasma.engg.nagoya-u.ac.jp

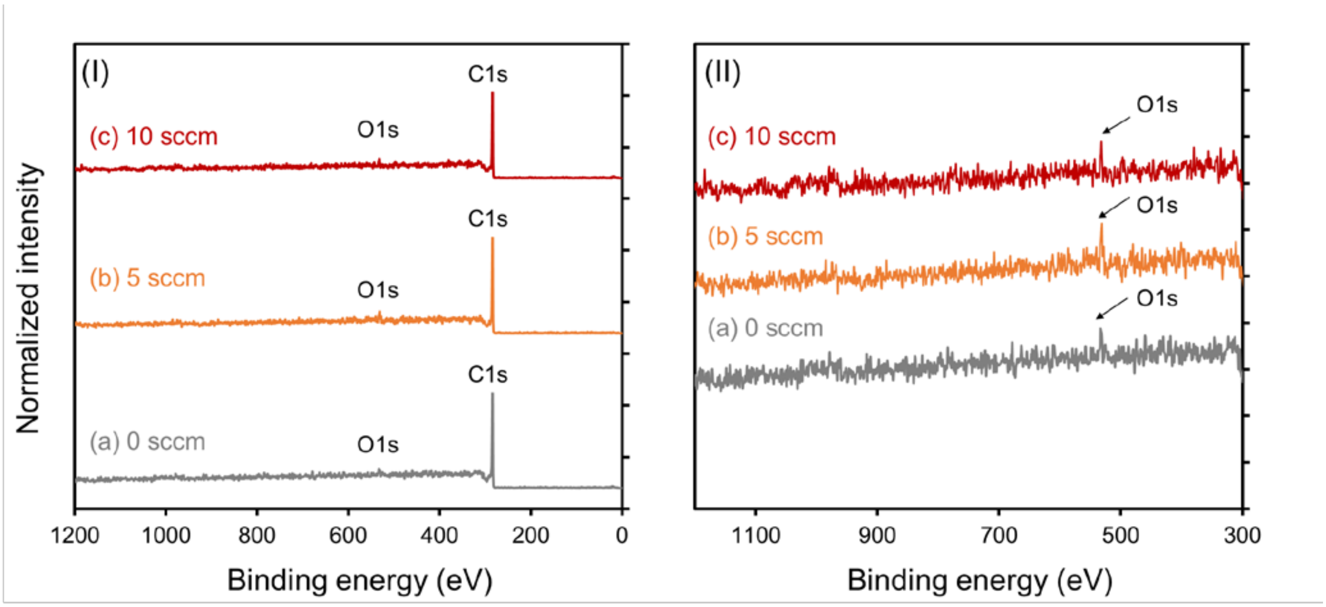

Figure S1. (I) the entire and (II) enlarged XPS spectra of (a) normal CNWs, (b) 5-sccm-O_2–, and (c) 10-sccm-O_2– high-quality CNWs. XPS spectra were normalized with respect to the C1s peak intensities.
